# Cooperative Interplay Between PGPR and *Trichoderma longibrachiatum* Reprograms the Rhizosphere Microecology for Improved Saline Alkaline Stress Resilience in Rice Seedlings

**DOI:** 10.3390/microorganisms13071562

**Published:** 2025-07-02

**Authors:** Junjie Song, Xueting Guan, Lili Chen, Zhouqing Han, Haojun Cui, Shurong Ma

**Affiliations:** Key Laboratory of Saline-Alkali Vegetation Ecology Restoration, Ministry of Education, Northeast Forestry University, Harbin 150040, China; songjunjie@nefu.edu.cn (J.S.); guanxt@nefu.edu.cn (X.G.); chenlili@nefu.edu.cn (L.C.); hzhouqq@163.com (Z.H.); m15776637683@163.com (H.C.)

**Keywords:** saline alkali soil amelioration, PGPR, *Trichoderma*-rhizobacteria synergy, rice growth promotion, soil microecology

## Abstract

Soil salinization has become a major obstacle to global agricultural sustainability. While microbial inoculants show promise for remediation, the functional coordination between *Trichoderma* and PGPR in saline alkali rhizospheres requires systematic investigation. Pot studies demonstrated that while individual inoculations of *Trichoderma longibrachiatum* (M) or *Bacillus aryabhattai* (A2) moderately improved rice growth and soil properties, their co-inoculation (A2 + M) synergistically enhanced stress tolerance and nutrient availability—increasing available nitrogen (AN +28.02%), phosphorus (AP +11.55%), and potassium (AK +8.26%) more than either strain alone, while more effectively mitigating salinity (EC −5.54%) and alkalinity (pH −0.13 units). High-throughput sequencing further revealed that the A2 + M treatment reshaped the rhizosphere microbiome, uniquely enriching beneficial taxa (e.g., Actinomycetota [+9.68%], Ascomycota [+50.58%], Chytridiomycota [+152.43%]), and plant-growth-promoting genera (e.g., *Sphingomonas*, *Trichoderma*), while drastically reducing saline-alkali-adapted Basidiomycota (−87.96%). Further analysis identified soil organic matter (SOM), AN, and AP as key drivers for the enrichment of Chytridiomycota and Actinomycetota, whereas pH and EC showed positive correlations with Mortierellomycota, Aphelidiomycota, unclassified_k__Fungi, and Basidiomycota. Collectively, the co-inoculation of *Trichoderma* and PGPR strains enhanced soil microbiome structure and mitigated saline alkali stress in rice seedlings. These findings demonstrate the potential of microbial consortia as an effective bio-strategy for saline alkali soil amelioration.

## 1. Introduction

Soil salinization is one of the major constraints affecting soil ecological functions and agricultural development [1]. Globally, approximately 800 million hectares of agricultural land are affected by salinity, with 20–33% of irrigated croplands experiencing varying degrees of salt stress [2]. Excessive salinity and sodium ions in soil induce particle dispersion, structural degradation, and reduced permeability [3,4]. Elevated salt and sodium ion concentrations significantly alter soil EC, pH, and exchangeable sodium percentage (ESP) [5,6,7]. The increased pH and EC directly impair soil microbial communities and enzyme activities, ultimately disrupting soil nutrient cycling and reducing nutrient availability [8]. The primary stresses imposed by high salinity on plants include osmotic stress and ion toxicity, which collectively inhibit mineral nutrient absorption and translocation, leading to substantial crop yield reduction [9,10]. The Songnen Plain in Northeast China, serving as a critical rice production region and one of the world’s three primary soda saline–alkali land distribution zones, encompasses 3.73 million hectares of saline–alkali soil with an annual increment of approximately 20,000 hectares of newly salinized land [11,12,13]. As a salt-sensitive crop, rice under saline–alkali stress exhibits ionic homeostasis disruption, photosynthetic efficiency decline, and ultimately demonstrates reduced biomass accumulation and yield depression [14].

Against the backdrop of severe challenges to global food security, the effective remediation of saline–alkali soils and the mitigation of their negative impacts on crop yields have emerged as key research priorities in agricultural and environmental sciences. Currently, three primary remediation approaches have been developed: physical, chemical, and biological methods [15]. Among these, microbial inoculants have garnered extensive research attention and application due to their eco-friendly nature and sustainable remediation potential [16,17]. Plant growth-promoting rhizobacteria (PGPR), as a common microbial inoculant, demonstrate significant value in improving soil physicochemical properties and enhancing plant stress resistance. PGPR can enhance soil nutrient availability through nitrogen fixation, phosphorus solubilization, and potassium mobilization, while also secreting phytohormones (e.g., indole-3-acetic acid [IAA], cytokinin [CTK]) to promote plant growth and boost antioxidant enzyme activities, thereby effectively alleviating abiotic stresses including salinity and drought [18,19,20,21]. Moreover, the application of microbial inoculants triggers complex interactions with indigenous rhizosphere microbial communities, influencing their diversity and composition. For instance, inoculation with *Bacillus paralicheniformis strain* SYN-191 reshaped the microbial community in ginger rhizosphere soil, reducing populations of pathogenic fungi (e.g., *Fusarium* sp.) while increasing beneficial bacterial groups (e.g., *Bacillus* sp. and *Pseudomonas* sp.) [22]. Under optimal nitrogen availability, rhizobia positively affected peanut growth and yield by remodeling the soil microbiome and metabolome [23]. Similarly, the addition of *Streptomyces* sp. *HU2014* altered wheat rhizosphere microbial composition and alleviated Cr(VI) stress [24].

*Trichoderma* spp., a group of beneficial fungi widely distributed in soils, have been recognized as a promising bioremediation strategy due to their remarkable potential in improving soil physicochemical properties and promoting plant growth. Research demonstrates that *Trichoderma* spp. can enhance soil quality and significantly reduce soil pH and EC by modifying the microbial community structure [25,26]. Furthermore, *Trichoderma* spp. establishes symbiotic relationships with plant seeds and rhizospheres, markedly improving plant resistance to both biotic and abiotic stresses through induced systemic resistance and enhanced antioxidant capacity [27,28]. Although many microorganisms show potential in ameliorating saline soils, single-strain inoculants often fail to comprehensively address the complex interplay of environmental factors such as soil pH, osmotic pressure, organic matter content, and salt concentration. In contrast, composite microbial agents (CMAs) exhibit superior remediation efficiency through synergistic interactions among constituent strains. These synergistic effects primarily manifest as mutual growth promotion and functional complementarity (e.g., biofilm co-assembly/substrate co-metabolism), thereby driving biogeochemical cycles and enhancing agricultural productivity and environmental remediation applications [29]. For example, the co-inoculation of rhizobia and PGPR in alfalfa (*Medicago sativa*) improved soil quality and enhanced plant tolerance to abiotic stress [30]. Similarly, the combined inoculation of arbuscular mycorrhizal fungi (AMF) and PGPR reduced soil EC and pH while increasing nutrient availability and the abundance of beneficial soil bacteria [31].

Current research demonstrates that the co-inoculation of *Trichoderma* spp. with various PGPR exhibits significant synergistic effects on plant growth promotion, nutrient uptake efficiency, and crop yield enhancement [32,33]. However, there are still very few studies on the synergistic mechanisms of this microbial community in improving plant tolerance to abiotic stress, especially regarding rhizosphere reprogramming—the dynamic reorganization of microbial networks under saline–alkali conditions [34]. Therefore, we specifically investigate how *Trichoderma*-PGPR interactions enhance rice seedling tolerance to saline–alkali stress through soil–plant–microbe crosstalk. We hypothesize that (1) the PGPR–*Trichoderma* synergy enhances rice physiological adaptation to saline–alkali stress; (2) co-inoculation significantly improves rhizosphere soil physicochemical properties; and (3) combined treatment restructures rhizosphere microbial communities by increasing beneficial taxa abundance. To test these hypotheses, this study aims to quantitatively evaluate the effects of co-inoculation on rice physiological characteristics and soil properties under saline–alkali stress, characterize the resulting changes in soil microbial community structure using high-throughput sequencing, and identify the key environmental factors driving microbial community assembly. Our findings will provide mechanistic insights into microbial interactions under saline–alkali stress, while offering practical guidance for developing effective microbial consortia in sustainable rice cultivation systems.

## 2. Materials and Methods

### 2.1. Preparation of Microbial Inoculants and Soil

The four salt–alkaline-tolerant plant growth-promoting bacterial strains used in this study were isolated from the rhizosphere soil of rice grown in saline–alkaline fields of Tailai County (46°23′26.9″ N, 123°24′33.6″ E), Heilongjiang Province, China. Strains were selected through functional screening for tolerance to high pH (pH 9.0) and salinity (7% NaCl), along with abilities to produce IAA and solubilize phosphate, including *Priestia megaterium* A1, *Bacillus aryabhattai* A2, *Pantoea pleuroti* B3, and *Enterobacter kobei* E2. All bacterial strains are preserved in 20% glycerol at −80 °C in our laboratory. The bacterial suspensions were prepared as follows: Each strain retrieved from −80 °C glycerol stocks was inoculated into LB liquid medium and cultured at 28 °C with shaking at 180 rpm until reaching the logarithmic growth phase (OD600 = 0.8–1). The cultures were centrifuged at 6000× *g* for 10 min (4 °C). After discarding the supernatant, the cell pellets were resuspended in an equal volume of sterile water for subsequent use.

The fungal strain *Trichoderma longibrachiatum* M (preservation number: CCTCC M 2021873) used in the experiment was kindly provided by the Life Science Laboratory of Northeast Forestry University. The strain is routinely maintained on potato dextrose agar (PDA) at 4 °C and preserved in 25% glycerol at −80 °C. For preparation, the strain from PDA slants was inoculated into PDB medium and cultured at 28 °C with shaking at 150 rpm for 7 days. The spore suspension was obtained by filtration through three layers of sterile gauze, followed by centrifugation at 6000× *g* for 10 min (4 °C). The spore concentration was adjusted to 1 × 10^8^ CFU/mL before use.

The saline–alkali soil was collected from the experimental base of Northeast Forestry University in Anda City, Heilongjiang Province, while the peat soil was purchased from the Flower Market in Harbin, Heilongjiang Province. Both soils were sieved through a 2 mm mesh and air-dried naturally. They were then uniformly mixed at a weight ratio of 1:1.5 (saline–alkali soil to peat soil). The basic physicochemical properties of the mixed soil are shown in Table S1. Each pot was filled with 1 kg of the mixed soil and prepared for subsequent experiments.

### 2.2. Pot Experiment

A completely randomized design was employed with 10 treatments: CK (control), M (*T. longibrachiatum*), A1 (*P. megaterium*), A2 (*B. aryabhattai*), B3 (*P. pleuroti*), E2 (*E. kobei*), and their respective co-inoculations (A1 + M, A2 + M, B3 + M, E2 + M), with five biological replicates per treatment (totaling 50 experimental units randomly arranged in the growth space). Prior to sowing, rice seeds were soaked for 3 h in respective bacterial suspensions (control seeds in sterile water). Pots were rotated daily to minimize positional effects. Ten days after sowing, seedlings were thinned to five uniformly sized plants per pot. To reinforce microbial colonization, 200 mL of the corresponding inoculum was applied twice per pot at five-day intervals, with soil moisture maintained at 80% field capacity throughout the experiment. Environmental conditions (temperature: 28 °C, relative humidity: 80%, light/dark cycle: 14/10 h) were monitored and maintained uniformly.

### 2.3. Plant Growth Parameters and Rhizosphere Soil Sampling

At 40 days of growth, from each treatment’s five pot-level biological replicates, one randomly selected plant per pot (totaling five plants per treatment) was used to measure plant height, root length, and shoot fresh/dry weight. Based on these growth parameters, four representative treatments (CK, A2, M, and A2 + M) showing the most significant and contrasting responses were selected for subsequent physiological and biochemical analyses.

For these selected treatments, leaf samples from one plant per pot across three randomly selected pots per treatment were used to determine antioxidant enzyme activities: catalase (CAT): H_2_O_2_ decomposition rate was measured at 240 nm using ultraviolet spectrophotometry; peroxidase (POD): absorbance changes in guaiacol oxidation products were measured at 470 nm; superoxide dismutase (SOD): NBT reduction inhibition rate was measured at 560 nm; and ascorbate peroxidase (APX): ascorbate oxidation rate was measured at 290 nm [35]. All measurements were performed using a spectrophotometer with three biological replicates. H_2_O_2_ content was determined using the potassium iodide (KI) method [36]. Malondialdehyde (MDA) content was measured by the thiobarbituric acid (TBA) method [37]. Each biochemical analysis included 3 technical replicates per sample to ensure measurement precision.

For the same selected treatments (CK, A2, M, and A2 + M), the rice plants were carefully removed from the pots, and their roots were gently shaken to dislodge loosely attached soil. Subsequently, rhizosphere soil samples were collected by brushing the root surfaces and tightly adhering soil with sterile brushes. These samples were immediately stored at −80 °C and later sent to Majorbio Co (Shanghai, China) for high-throughput sequencing analysis (16S rRNA and ITS sequencing) to determine the bacterial and fungal community composition and diversity. Following this, surface soil samples (0–10 cm depth) were collected from the same sampling area. After thorough mixing, the soil was air-dried and sieved through a 2 mm mesh for subsequent analysis of soil physicochemical properties.

### 2.4. Soil Physicochemical Property Analysis

Soil pH was measured using a pH meter (FE28, METTLER-TOLEDO, Columbus, OH, USA) at a soil-to-water ratio of 1:2.5 (*m*/*v*). Soil electrical conductivity (EC) was determined using a conductivity meter (Bante902P, Bante, China) at a soil-to-water ratio of 1:5 (*m*/*v*) [3]. Soil Organic Matter (SOM) was determined using the potassium dichromate oxidation-external heating method [38]. Alkali-hydrolyzable nitrogen was measured by the alkaline hydrolysis diffusion method [39]. Available phosphorus was extracted with sodium bicarbonate (NaHCO_3_) and quantified by molybdenum-antimony anti-spectrophotometry [40]. Exchangeable potassium was extracted with ammonium acetate (NH_4_OAc) and measured using flame photometry [41]. All analyses were performed with three biological replicates to ensure data accuracy and reliability.

### 2.5. DNA Extraction, Amplicon Sequencing, and Data Processing

Soil microbial genomic DNA was extracted using the E.Z.N.A.^®^ Soil DNA Kit (Omega Bio-tek, Norcross, GA, USA). DNA quality was assessed by 1% agarose gel electrophoresis, and concentration/purity was determined using a NanoDrop 2000 UV-vis spectrophotometer. Amplicon sequencing of bacterial 16S rRNA and fungal ITS regions was performed by Majorbio Co., Ltd. (Shanghai, China). The V3-V4 region of bacterial 16S rRNA was amplified using primers 338F (5′-ACTCCTACGGAGGCAGCAG-3′) and 806R (5′-GGACTACHVGGGTWTCTAAT-3′). The fungal ITS region was amplified with primers ITS1F (5′-CTTGTCATTTAGAGGAAGTAAGTAA-3′) and ITS2R (5′-GCTGCGTTCTTCATCGATGC-3′). Three replicate PCR reactions were performed per sample. Paired-end reads were processed using FLASH v1.2.11 for merging and quality control. After removing singletons, high-quality sequences were clustered into operational taxonomic units (OTUs) at 97% similarity using the UPARSE algorithm, with chimera removal during clustering. Taxonomic assignment was performed against the SILVA database (available online: http://www.arb-silva.de, accessed on 1 July 2025) for bacteria and the UNITE fungal database (available online: http://unite.ut.ee/index.php, accessed on 1 July 2025) for fungi.

### 2.6. Data Analysis

Microbial sequence processing was performed on the Majorbio Cloud Platform (available online: https://www.majorbio.com, accessed on 1 July 2025) using Mothur (v1.45.1) for both 16S rRNA and ITS datasets. Differences in microbial α-diversity indices (Chao1, Shannon, Simpson, and Sobs) were analyzed using one-way ANOVA with post hoc tests. Sequencing depth was validated by constructing Shannon index rarefaction curves using R software (v3.3.1). Beta diversity analysis was conducted in QIIME2 (v2020.2.0) by computing Bray Curtis distance matrices, followed by principal coordinate analysis (PCoA) to reveal community β-diversity patterns. Inter-group differences were assessed using ANOSIM (999 permutations, *p* < 0.05). LEfSe analysis was employed to identify biomarker taxa across treatment groups. Redundancy analysis (db-RDA) based on Bray Curtis distance matrices was performed to examine correlations between microbial community structure and soil physicochemical properties. Treatment effects on rice physiological/biochemical parameters and soil properties were analyzed by one-way ANOVA followed by Fisher’s Least Significant Difference (LSD) post hoc tests (α = 0.05) using SPSS 26.0

## 3. Results

### 3.1. Effects of Microbial Inoculation on Rice Physiological and Biochemical Parameters

As shown in Table 1, different inoculation treatments significantly affected rice growth parameters under saline–alkali stress. Compared with CK, all inoculation treatments significantly alleviated the inhibitory effects of saline–alkali stress on rice growth (Figure S1). The treated rice plants exhibited compact plant architecture and dark green leaves, while the CK plants showed typical symptoms of saline–alkali stress, including leaf yellowing and curling. In terms of growth parameters, all inoculation treatments significantly increased plant height, root length, dry weight, and fresh weight to varying degrees (*p* < 0.05). Under saline–alkali conditions, dual inoculation treatments generally exhibited stronger growth-promoting effects than single inoculation treatments. Among them, the A2 + M treatment showed the most significant alleviation effect (Table 1), increasing plant height, root length, dry weight, and fresh weight by 67.75%, 65.60%, 211.42%, and 190.92%, respectively, compared to CK. However, it is noteworthy that among the dual inoculation treatments, the E2 + M treatment, although still demonstrating some growth-promoting effects compared to CK, was less effective than the corresponding single inoculation treatments (M and E2). This may be attributed to antagonistic interactions between microbial strains under saline–alkali conditions. Based on these morphological observations and growth parameter analyses, we selected the most effective treatment combinations (CK, M, A2, and A2 + M) for subsequent experiments.

**Figure 1:**
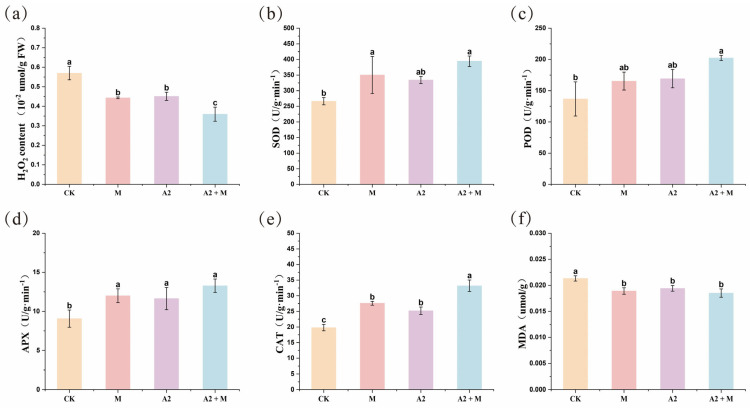
Microbial inoculant application enhanced rice tolerance to saline–alkali stress. (**a**) H_2_O_2_ content. (**b**) SOD (superoxide dismutase) activity. (**c**) POD (peroxidase) activity. (**d**) APX (ascorbate peroxidase) activity. (**e**) CAT (catalase) activity. (**f**) MDA content. Data are derived from three biological replicates (*n* = 3) and presented as mean ± standard error (SE). Different lowercase letters indicate statistically significant differences (*p* < 0.05) among treatments.

Under saline-alkaline stress conditions, plants activate their antioxidant enzyme systems to scavenge excess reactive oxygen species (ROS), thereby mitigating oxidative damage to cellular components. Our results demonstrate that microbial inoculation treatments significantly enhanced the activities of key antioxidant enzymes (POD, SOD, APX, and CAT) in rice leaves (Figure 1b–e). Notably, the A2 + M dual-inoculation treatment exhibited the most pronounced stimulatory effects, with POD, SOD, APX, and CAT activities increased by 47.95%, 48.06%, 46.46%, and 67.68%, respectively, compared to CK (Table S2). Corresponding analysis of oxidative stress markers revealed that the A2 + M treatment significantly reduced H_2_O_2_ and MDA contents by 36.93% and 13.18%, respectively, relative to CK (Figure 1a,f; Table S2). These findings align well with the observed upregulation of antioxidant enzyme activities, suggesting that the A2 + M treatment effectively strengthens the rice antioxidant defense system, thereby alleviating saline–alkaline stress-induced oxidative damage.

### 3.2. Effects of Microbial Inoculation on Rhizosphere Soil Physicochemical Properties

**Figure 2:**
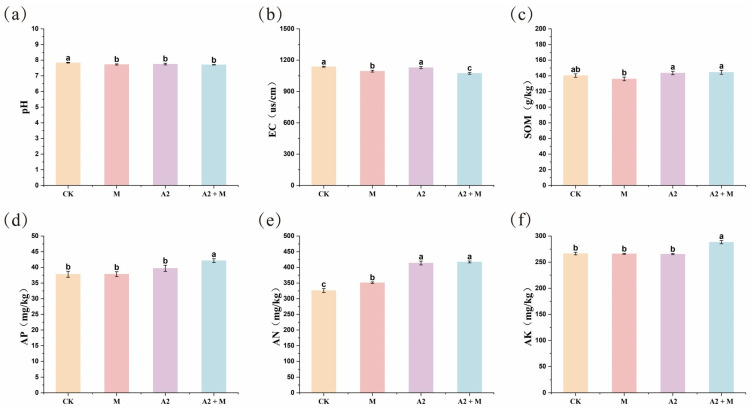
Effects of different inoculation treatments on physicochemical properties of rice rhizosphere soil. (**a**) pH. (**b**) Electrical conductivity (EC). (**c**) Soil organic matter (SOM). (**d**) Available phosphorus (AP). (**e**) Alkali-hydrolyzable nitrogen (AN). (**f**) Available potassium (AK). Data represent mean ± standard error (SE) from three biological replicates (*n* = 3). Different lowercase letters indicate significant differences among treatments (*p* < 0.05).

All inoculation treatments significantly altered the physicochemical properties of rice rhizosphere soil (Figure 2; Table S3). Both single inoculation (M or A2 alone) and co-inoculation (A2 + M) reduced soil pH and electrical conductivity (EC), with the co-inoculation treatment showing the most pronounced effects (*p* < 0.05). In addition, microbial inoculation modified soil nutrient contents. Compared to the control (CK), the co-inoculation treatment (A2 + M) significantly increased soil organic matter (SOM), available phosphorus (AP), available nitrogen (AN), and available potassium (AK) in the rice rhizosphere (*p* < 0.05). Notably, the co-inoculation treatment resulted in higher soil nutrient levels than single inoculation treatments, demonstrating that the combined application had greater effects than individual inoculations. To investigate the relationship between plant dry weight and soil physicochemical properties, Pearson correlation analysis was performed. As shown in Figure S2, rice dry weight exhibited strong positive correlations with both AP and AK, suggesting that elevated phosphorus and potassium levels in soil may promote plant biomass accumulation.

### 3.3. Effects of Different Inoculation Treatments on Rhizosphere Microbial Community Diversity

Sequencing analysis generated 852,750 high-quality bacterial sequences (average length: 415 bp) and 773,303 fungal sequences (average length: 254 bp) across all treatments using the Illumina MiSeq platform. Rarefaction curves (Figure S3) plateaued for all samples, indicating sufficient sequencing depth for reliable analysis.

**Figure 3:**
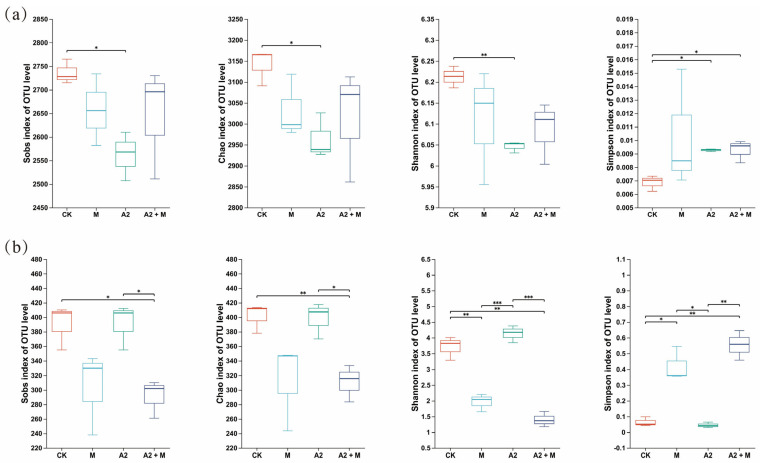
Microbial community diversity under different inoculation treatments. Alpha diversity indices for (**a**) soil bacteria and (**b**) soil fungi. *, **, and *** indicate significant differences among treatments at *p* < 0.05, *p* < 0.01, and *p* < 0.001, respectively.

**Figure 4:**
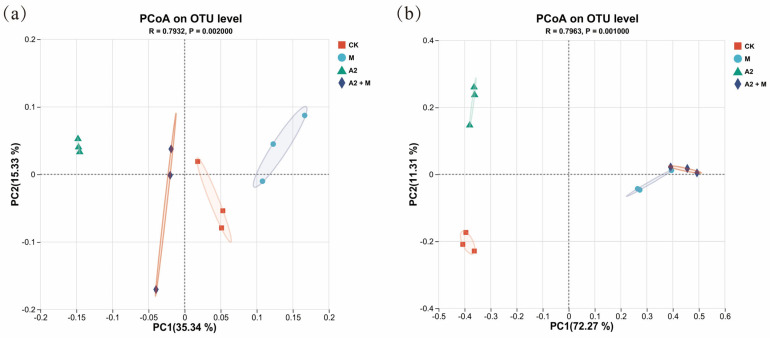
Beta diversity based on PCoA analysis for (**a**) bacterial and (**b**) fungal communities.

Microbial community α-diversity was assessed using Sobs, Chao1, Simpson, and Shannon indices. For bacterial communities (Figure 3a), A2 single inoculation significantly reduced both diversity and richness compared to CK (*p* < 0.05), while other treatments showed no significant differences. Regarding fungal communities (Figure 3b), A2 + M co-inoculation significantly decreased richness and diversity versus CK (*p* < 0.05). Additionally, M single inoculation significantly reduced fungal richness indices (Sobs and Chao1; *p* < 0.05). Principal coordinates analysis (PCoA) based on Bray Curtis distances revealed distinct microbial community structures among treatments (Figure 4a,b). For bacterial communities, PC1 and PC2 explained 35.34% and 15.33% of the variation, respectively. Fungal community variation was primarily explained by PC1 (72.27%) and PC2 (11.31%). Significant separation (*p* < 0.01) was observed among all treatments (CK, M, A2, and A2 + M), indicating pronounced differences in rhizosphere microbial composition.

### 3.4. Effects of Inoculation Treatments on Rhizosphere Microbial Community Structure

**Figure 5:**
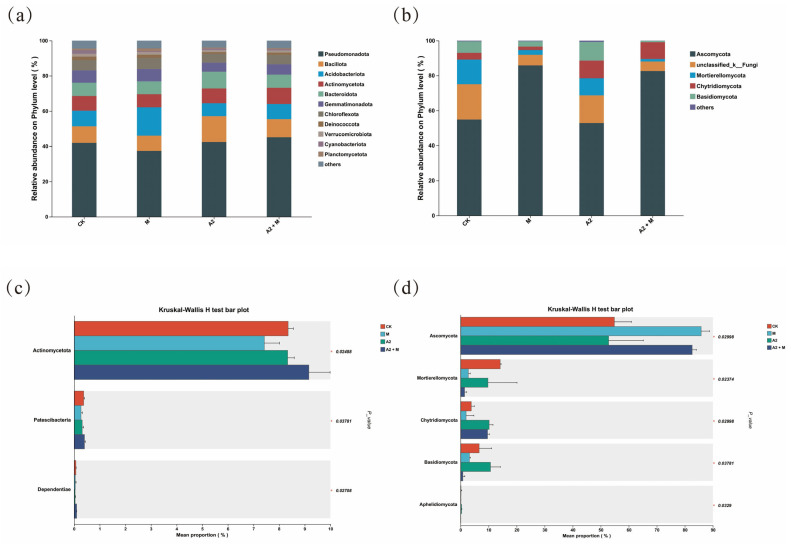
Effects of different inoculation treatments on the composition of bacterial (**a**,**c**) and fungal (**b**,**d**) phyla in rice rhizosphere soil.

The bacterial communities were dominated by 11 phyla with relative abundance >1%, including Pseudomonadota, Bacillota, Acidobacteriota, Actinomycetota, Bacteroidota, Gemmatimonadota, Chloroflexota, Deinococcota, Verrucomicrobiota, Cyanobacteriota, and Planctomycetota, which collectively accounted for >95% of total bacterial sequences (Figure 5a). For fungal communities, five phyla showed relative abundance >1%: Ascomycota (>50% across all treatments), unclassified_k__Fungi, Mortierellomycota, Chytridiomycota, and Basidiomycota (Figure 5b). The Kruskal Wallis H test revealed significant differences in phylum-level composition among treatments (Figure 5c,d). Compared with CK, the dual inoculation treatment (A2 + M) significantly increased the relative abundances of Actinomycetota, Patescibacteria, Dependentiae, Ascomycota, and Chytridiomycota, while decreasing Mortierellomycota and Basidiomycota (*p* < 0.05). These results indicate that microbial inoculation can significantly alter the relative abundances of dominant phyla in the microbial communities.

**Figure 6:**
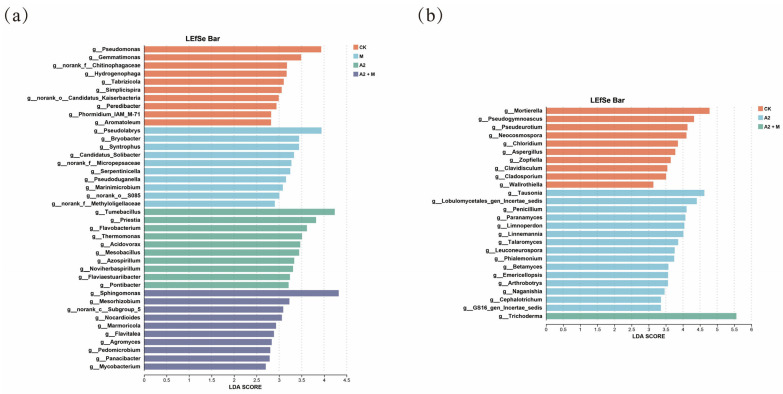
Identification of characteristic dominant microbial genera in different saline–alkali soil treatments using LEfSe analysis. (**a**) Bacterial communities. (**b**) Fungal communities.

LEfSe analysis was performed to identify significantly enriched microbial taxa among different treatments, using an LDA score threshold >3.5. For bacterial communities (Figure 6a), the M treatment specifically enriched *Pseudolabrys*, while the A2 treatment enriched *Flavobacterium*, *Priestia*, *Tumebacillus*, and *Thermomonas*. The A2 + M co-inoculation uniquely enriched *Sphingomonas*. In fungal communities (Figure 6b), the A2 + M treatment specifically enriched *Trichoderma*, whereas no significant taxa were enriched in the M treatment alone. These distinct microbial distribution patterns reflect the differential effects of microbial inoculation on rhizosphere microbial populations.

### 3.5. Interactions of Rice Rhizosphere Microbial Communities with Environmental Variables and Physiology

**Figure 7:**
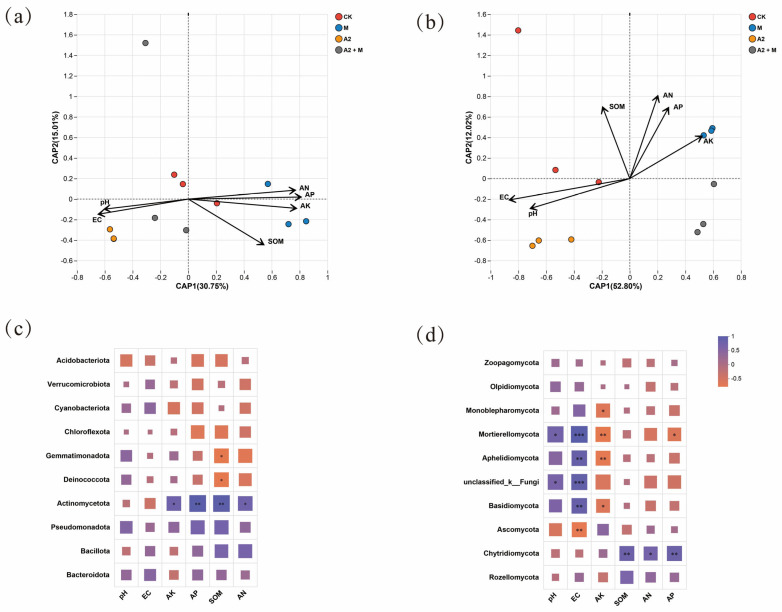
Interactions between environmental factors and microbial communities. (**a**) db-RDA of bacterial communities. (**b**) db-RDA of fungal communities. (**c**) Heatmap showing correlations between the top 10 bacterial phyla and environmental factors. (**d**) Heatmap showing correlations between the top 10 fungal phyla and environmental factors. Significance levels: * *p* < 0.05, ** *p* < 0.01, *** *p* < 0.001.

Distance-based redundancy analysis (db-RDA) and correlation heatmap analysis revealed significant associations between environmental factors and microbial communities (Figure 7). The db-RDA explained 45.76% and 64.82% of the total variance for bacterial and fungal communities, respectively, confirming the reliability of the analysis. The addition of microbial inoculants significantly altered soil microbial community structure. In bacterial communities, only Actinomycetota showed significant positive correlations with soil nutrients (SOM, AP, AN, and AK; *p* < 0.05), which was consistent with the trends observed in the A2 + M treatment. For fungal communities, SOM, AN, and AP were positively correlated with Chytridiomycota (*p* < 0.05), while pH and EC were positively associated with Mortierellomycota, Aphelidiomycota, unclassified_k__Fungi, and Basidiomycota. These results demonstrate that environmental factors differentially influence fungal and bacterial communities.

Spearman correlation analysis revealed significant associations between rice microbial community structure and physicochemical properties (Figure S4). Actinomycetota, Ascomycota, and Chytridiomycota showed positive correlations with antioxidant enzyme activities, while Cyanobacteriota, unclassified_k__Fungi, Mortierellomycota, and Basidiomycota exhibited negative correlations. These results suggest that specific microbial taxa may participate in regulating the rice antioxidant defense system, with Actinomycetota, Ascomycota, and Chytridiomycota potentially promoting antioxidant enzyme production, while Cyanobacteriota and others may compete with or inhibit this process.

## 4. Discussion

### 4.1. Effects of Microbial Inoculation on Rice Physiological and Biochemical Characteristics

Soil salinization significantly inhibits plant growth and development through ionic toxicity and oxidative stress [42]. Under such stress conditions, PGPR and *Trichoderma* inoculation demonstrate remarkable growth-promoting effects, with the combined application of PGPR and *Trichoderma* showing the most pronounced benefits. This indicates their synergistic effects on rice growth in saline–alkali soils, which aligns with multiple studies reporting that *Trichoderma* treatment significantly enhances biomass accumulation in rice, wheat, and maize under salt stress [43,44,45]. Notably, Jambhulkar et al. [46] found that the co-inoculation of *Trichoderma harzianum* and *Pseudomonas fluorescens* not only reduced rice blast incidence but also promoted plant height and yield, further confirming the synergistic interactions between microorganisms in plant growth promotion.

From a molecular perspective, saline–alkali stress induces excessive ROS accumulation in plants, leading to oxidative damage. To counteract this, plants have evolved a sophisticated ROS scavenging system, including enhanced synthesis of non-enzymatic antioxidants (e.g., ascorbic acid and glutathione) and activation of antioxidant enzymes (e.g., SOD, POD, and CAT) [47,48,49]. Studies show that Trichoderma significantly increases the activity of key antioxidant enzymes (GR, GST, SOD, POD, and CAT) in host plants, thereby alleviating ROS-induced oxidative damage [49]. Similarly, PGPR can mitigate oxidative stress by secreting antioxidant enzymes or regulating ROS-scavenging gene expression [50]. In this study, rice leaves treated with A2 (PGPR), M (*Trichoderma*), and A2 + M (co-inoculation) exhibited significantly elevated antioxidant enzyme activities (*p* < 0.05) (Figure 1), consistent with previous findings.

PGPR and *Trichoderma* employ complementary mechanisms to alleviate saline alkali stress: PGPR primarily promotes plant growth directly through the secretion of IAA, ACC deaminase, and siderophores [50], whereas *Trichoderma* indirectly enhances stress tolerance by activating systemic resistance (e.g., inducing antioxidant enzymes) and improving the rhizosphere microenvironment [51]. When co-inoculated, they establish a unique “metabolic complementarity” effect—PGPR’s phosphate solubilization and nitrogen fixation synergize with Trichoderma’s organic acid secretion to increase soil-available nitrogen and phosphorus [34,52]. Meanwhile, Trichoderma-induced root expansion further enlarges the absorption interface for PGPR-derived growth-promoting substances [53]. These integrated regulatory mechanisms ultimately lead to a significant increase in rice biomass.

### 4.2. Effects of Microbial Inoculation on Soil Nutrients

Soil nutrients serve as fundamental indicators for assessing soil fertility status. Numerous studies have demonstrated that introducing beneficial microorganisms into saline–alkali soils can significantly improve soil nutrient conditions, thereby promoting plant growth [31,54,55]. Among these factors, soil pH represents a key determinant of soil health [56]. Previous research has confirmed that microbial inoculants containing *Trichoderma* can effectively reduce soil pH, alleviate alkalization in the rhizosphere soil of alfalfa, and significantly increase its biomass [57]. Similarly, this study found that co-inoculation of *Trichoderma* and PGPR effectively mitigated soil alkalization and significantly lowered soil pH (Figure 2), which is highly consistent with previous research findings.

From a mechanistic perspective, *Trichoderma* and PGPR synergistically improve soil environment through the following pathways: (1) organic acid-mediated nutrient activation: *Trichoderma* secretes organic acids such as oxalic acid and citric acid to effectively dissolve insoluble phosphates and mineral-bound nitrogen, significantly enhancing the bioavailability of phosphorus and nitrogen in soil [58,59]; (2) microbial nitrogen fixation: Certain PGPR strains possess nitrogenase activity, enabling the conversion of atmospheric nitrogen into plant-available ammonium nitrogen [60]; and (3) mineral weathering: PGPR promotes silicate mineral weathering through acidification, releasing soluble potassium while dissolving fixed phosphates [61].

Notably, although the AP and AN contents in soil treated with *Trichoderma* alone were significantly higher than those in soil treated with PGPR alone, the latter exhibited superior performance in terms of rice plant height and root length growth. This phenomenon may be attributed to several factors: the high nutrient use efficiency of *Trichoderma*—by enhancing root exudation and nutrient absorption kinetics, *Trichoderma* accelerates plant assimilation of soil available nutrients, resulting in relatively lower AP/AN contents in the soil [62]; the synergistic effect of PGPR—although PGPR is less effective in nutrient activation, it may indirectly promote nutrient cycling by improving rhizosphere microbial community structure.

The research results demonstrate that despite the lower available nutrient content in soil treated with *Trichoderma*, it still significantly increases rice biomass, indicating that the primary role of *Trichoderma* lies in optimizing nutrient use efficiency rather than simply activating nutrients. This finding provides a new perspective for understanding microbe–plant interactions, suggesting that microbe-mediated nutrient cycling processes are more decisive for plant productivity than static nutrient pools. Future research should focus on analyzing the spatiotemporal dynamics of nutrient flow under co-inoculation conditions.

### 4.3. Effects of Microbial Inoculation on Soil Microbial Diversity and Composition

Soil microbial communities, as core biological components for maintaining ecosystem functions, directly influence soil quality and plant productivity through their structure and functional diversity [63]. This study found that different microbial inoculation treatments differentially affected rice rhizosphere microbial community structure. Specifically, the PGPR single inoculation (A2) significantly reduced bacterial α-diversity indices, while *Trichoderma* spp. inoculations (single M and dual A2 + M) significantly decreased fungal α-diversity indices (Figure 3). Existing studies demonstrate the dual effects of exogenous microbial inoculants on rhizosphere microecology. Some studies confirm that inoculants can significantly promote plant root microbial community development and increase rhizosphere soil microbial α-diversity [64,65,66]. However, other research has found that although microbial inoculants effectively promote plant growth, they may reduce rhizosphere microbial diversity [67,68]. This reduction effect may originate from the introduction of high-density single-strain inoculants that alter the original microecological balance through resource competition and niche preemption mechanisms, causing indigenous microbial populations with weaker adaptability to decline due to their inability to rapidly respond to environmental stress [66]. This competitive exclusion phenomenon ultimately manifests as reduced soil microbial community richness and diversity.

The dual inoculation treatment (A2 + M) significantly increased the relative abundances of Actinomycetota, Planctomycetota, Ascomycota, and Chytridiomycota (*p* < 0.05), while decreasing Mortierellomycota and Basidiomycota abundances (*p* < 0.05) (Figure 5). Actinomycetota, as key functional microorganisms, possess both organic matter degradation and antimicrobial activities [69]. Their abundance positively correlates with resource availability as typical copiotrophs [70], consistent with our findings showing significant positive correlations between Actinomycetota relative abundance and AP, AN, AK, and SOM contents (*p* < 0.05) (Figure 7). The increased dominance of Ascomycota may relate to their organic matter decomposition and stress tolerance capabilities in saline–alkali environments [71]. Basidiomycota, exhibiting strong salt resistance, are dominant in saline soils and showed a significant positive correlation with EC (*p* < 0.05) [72]. LEfSe analysis revealed treatment-specific functional microbial taxa: the dual inoculation significantly enriched plant-growth-promoting *Sphingomonas* (bacteria) and *Trichoderma* (fungi). These microbes likely synergistically alleviate saline–alkali stress—*Sphingomonas* through direct phytohormone production and *Trichoderma* via indirect mechanisms like soil enzyme activation and nutrient availability enhancement [49,73]. These results demonstrate that microbial inoculants not only alter rhizosphere community composition but may also improve plant stress adaptation by regulating specific functional microbe abundances.

## 5. Conclusions and Future Perspectives

In this study, we investigated the effects of the combined application of *Trichoderma* and PGPR on rice growth, soil nutrients, and rhizosphere microbial communities through pot experiments. The results demonstrated that the co-inoculation of PGPR and *Trichoderma* significantly improved soil physicochemical properties, including enhanced availability of soil nutrients, reduced salinity, and improved physiological and biochemical responses in rice, such as increased antioxidant enzyme activity and decreased malondialdehyde (MDA) content. At the microbial level, the combined treatment promoted the enrichment of beneficial microbial taxa and optimized the rhizosphere microbial community structure. Overall, the application of *Trichoderma* and PGPR improved soil microbial composition and effectively alleviated the negative impacts of saline–alkali stress on rice. These findings suggest that the synergistic use of *Trichoderma* and PGPR holds significant potential for saline–alkali soil remediation and sustainable agricultural practices.

This study provides a theoretical foundation and technical support for microbial remediation of saline–alkali soils. Future research will focus on the following key directions: (1) developing preparation techniques for compound microbial inoculants suitable for large-scale production, addressing technical bottlenecks such as strain compatibility and viability maintenance; (2) conducting 3–5 year fixed-position field trials in soda saline-alkali soil regions of the Songnen Plain to validate long-term effects of the inoculants; (3) elucidating the molecular mechanisms underlying Trichoderma–PGPR synergism through multi-omics approaches; and (4) establishing precision application protocols based on soil type characterization.

## Figures and Tables

**Table 1:** Effects of different inoculation treatments on rice seedling biomass under saline alkali stress.

Treatments	Plant Height (cm)	Root Length (cm)	Dry Weight (g)	Fresh Weight (g)
CK	23.69 ± 1.63 f	5.93 ± 1.13 e	0.06 ± 0.01 f	0.32 ± 0.10 d
M	33.41 ± 1.72 c	7.62 ± 0.90 d	0.09 ± 0.02 de	0.54 ± 0.14 b
A1	29.80 ± 0.55 e	7.93 ± 0.68 cd	0.09 ± 0.01 de	0.54 ± 0.06 b
A2	30.89 ± 0.95 de	7.31 ± 0.84 de	0.08 ± 0.02 e	0.49 ± 0.10 bc
B3	32.09 ± 1.06 cd	8.10 ± 1.83 cd	0.11 ± 0.03 cd	0.57 ± 0.15 b
E2	32.86 ± 0.82 c	8.29 ± 1.28 bcd	0.09 ± 0.01 de	0.56 ± 0.07 b
A1 + M	35.38 ± 1.18 b	11.43 ± 2.53 a	0.13 ± 0.02 bc	0.81 ± 0.12 a
A2 + M	39.74 ± 1.44 a	9.82 ± 0.48 b	0.18 ± 0.03 a	0.93 ± 0.12 a
B3 + M	36.61 ± 2.44 b	9.35 ± 0.40 bc	0.14 ± 0.03 b	0.89 ± 0.18 a
E2 + M	29.92 ± 1.30 e	7.56 ± 1.20 d	0.07 ± 0.01 ef	0.40 ± 0.06 cd

Note: Data are presented as mean ± standard error (SE) from three biological replicates (*n* = 5). Different lowercase letters indicate statistically significant differences (*p* < 0.05). Treatments: Single inoculations: CK (control), M (*Trichoderma longibrachiatum*), A1 (*Priestia megaterium*), A2 (*Bacillus aryabhattai*), B3 (*Pantoea pleuroti*), E2 (*Enterobacter kobei*). Co-inoculations: A1 + M (*P. megaterium* + *T. longibrachiatum*), A2 + M (*B. aryabhattai* + *T. longibrachiatum*), B3 + M (*P. pleuroti* + *T. longibrachiatum*), E2 + M (*E. kobei* + *T. longibrachiatum*).

## Data Availability

The data presented in this study are available on request from the corresponding author.

## Supplementary Materials

The following supporting information can be downloaded at: https://www.mdpi.com/article/10.3390/microorganisms13071562/s1, Figure S1: Effect of microbial inoculation on rice morphology; Figure S2: Correlation analysis between rice dry weight (DW) and soil (AN, AP, AK); Figure S3: Rarefaction curves based on Shannon index for bacterial and fungal communities under different treatments; Figure S4: Correlation analysis of rice physicochemical properties with bacterial and fungal phyla; Table S1: Basic physicochemical properties of the experimental soils.; Table S2: Effects of microbial inoculation on physiological indices of rice stress resistance; Table S3: Effects of microbial inoculation on physicochemical properties of rice rhizosphere soil.
