# Supplementary material for: Cooperative Interplay Between PGPR and Trichoderma longibrachiatum Reprograms the Rhizosphere Microecology for Improved Saline Alkaline Stress Resilience in Rice Seedlings
Source: Microorganisms. 2025 Jul 2;13(7):1562. doi: 10.3390/microorganisms13071562 (PMC12298565; doi:10.3390/microorganisms13071562)
Supplement: Supplementary file 1 [file microorganisms-13-01562-s001.zip › microorganisms-3681869-supplementary.pdf]

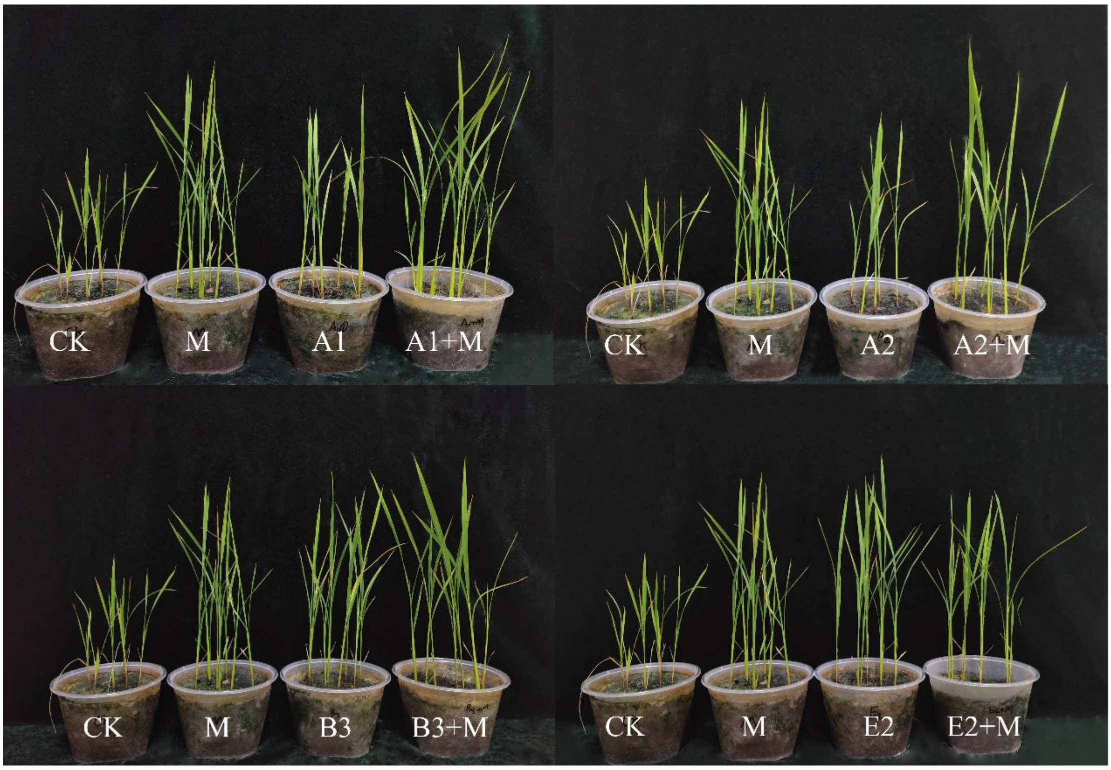

Figure S1 Effect of microbial inoculation on rice morphology

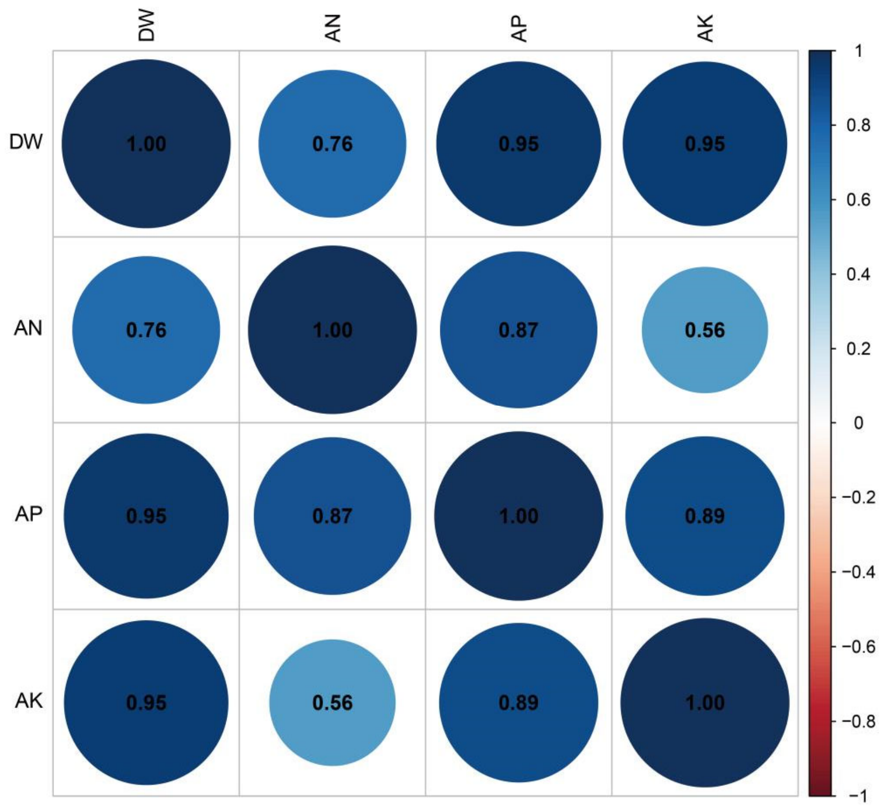

Figure S2 Correlation analysis between rice dry weight (DW) and soil (AN, AP, AK). The size and color of the circles indicate the strength of the correlation coefficients.

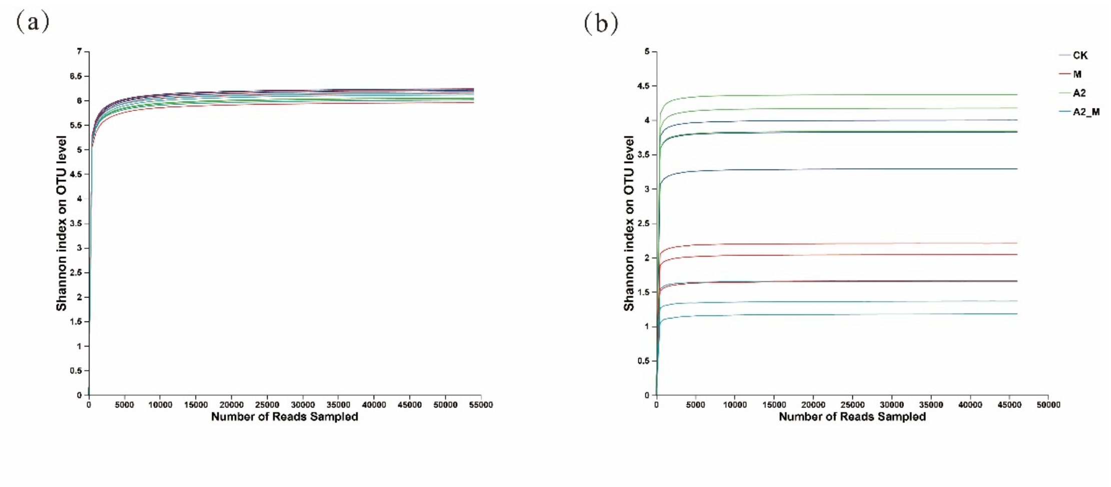

Figure S3 Rarefaction curves based on Shannon index for bacterial (a) and fungal (b) communities under different treatments.

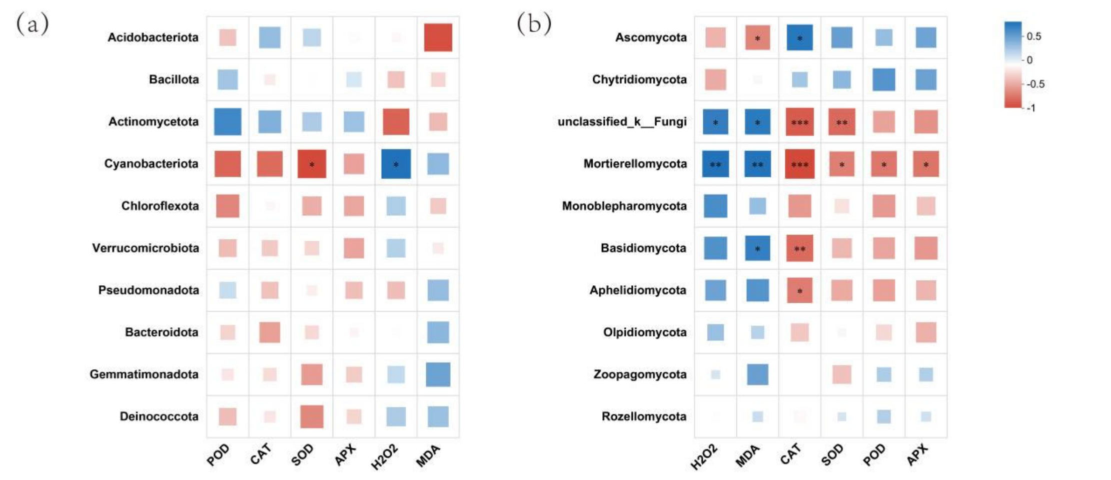

Figure S4 Correlation analysis of rice physicochemical properties with bacterial (a) and fungal (b) phyla. Significance levels: *$p < 0.05$, **$p < 0.01$, ***$p < 0.001$.

Table S1. Basic physicochemical properties of the experimental soils.

	pH	EC（ms/cm）	SOM （g/kg）	TN （g/kg）	TP （g/kg）	TK （g/kg）
Peat soil	5.91	0.53	200.07	7.4	0.94	12.30
Saline-alkali soil	10.82	3.78	14.33	0.75	0.37	18.17
Mixed soil	7.92	1.37	143.30	4.88	0.50	14.27

Table S2 Effects of microbial inoculation on physiological indices of rice stress resistance

	CK	M	A2	A2+M
H2O2 （10-2 umol/g FW）	0.5702 ± 0.0346 a	0.4435 ± 0.0045 b	0.4512 ± 0.0212 b	0.3596 ± 0.0358 c

SOD （U/g·min-1）	266.32 ± 11.62 b	350.52 ± 59.45 a	334.19 ± 11.62 ab	394.33 ± 16.90 a
POD （U/g·min-1）	136.80 ± 27.38 b	165.40 ± 14.47 ab	169.20 ± 14.66 ab	202.40 ± 4.26 a
APX （U/g·min-1）	9.07 ± 1.10 b	12.00 ± 0.89 a	11.64 ± 1.43 a	13.29 ± 0.86 a
CAT （U/g·min-1）	19.8 ± 1.04 c	27.60 ± 0.60 b	25.2 ± 1.20 b	33.2 ± 1.83 a
MDA （umol/g）	0.0213 ± 0.0005 a	0.0189 ± 0.0006 b	0.0194 ± 0.0006 b	0.0185 ± 0.0008 b

Table S3 Effects of microbial inoculation on physicochemical properties of rice rhizosphere soil.

	**CK**	**M**	**A2**	**A2+M**
**pH**	7.84 ± 0.02 a	7.73 ± 0.04 b	7.75 ± 0.05 b	7.71 ± 0.02 b
**EC（us/cm）**	1137 ± 4.36 a	1094.67 ± 8.5 b	1129 ± 11.27 a	1074 ± 8.89 c
**SOM（g/kg）**	140.27 ± 2.22 ab	135.97 ± 2.36 b	143.57 ± 2.21 a	144.5 ± 2.52 a
**AP（mg/kg）**	37.8 ± 0.95 b	37.83 ± 0.76 b	39.7 ± 0.98 b	42.17 ± 0.6 a
**AN（mg/kg）**	326 ± 6.24 c	351 ± 2.65 b	414 ± 6.24 a	417.33 ± 3.06 a
**AK（mg/kg）**	266.33 ± 2.52 b	266 ± 1 b	265.33 ± 1.15 b	288.33 ± 3.06 a
